# Didymin, a natural flavonoid, relieves the progression of myocardial infarction via inhibiting the NLR family pyrin domain containing 3 inflammasome

**DOI:** 10.1080/13880209.2022.2148170

**Published:** 2022-11-23

**Authors:** Yong Zhang, GuLi RuXian

**Affiliations:** a Department of Cardiology, First Affiliated Hospital, School of Medicine, Shihezi University, Shihezi, China; b Department of Digestive Internal Medicine, School of Medicine, First Affiliated Hospital, Shihezi University, Shihezi, China

**Keywords:** Ischaemia–reperfusion, NOD-like receptor protein 3, fibrosis

## Abstract

**Context:**

Globally, the morbidity and mortality of cardiovascular diseases remain high. Didymin, a flavonoid glycoside, has long been used as a dietary antioxidant.

**Objective:**

To determine the role of didymin in myocardial infarction (MI), and its possible myocardial protective mechanism.

**Materials and methods:**

C57/BL6 mice (aged 6–8 weeks, *n* = 40) were divided into five groups: sham group, ischaemia–reperfusion (I/R) group, I/R + didymin (1 mg/kg) group, I/R + didymin (2 mg/kg) group and I/R + didymin (4 mg/kg) group. Didymin was administered intragastrically daily before I/R for 5 consecutive days. H9C2 cells were divided into five groups: control group, H/R group, H/R + didymin (3 μM) group, H/R + didymin (10 μM) group and H/R + didymin (30 μM) group. H9C2 cells were treated with didymin for 24 h before hypoxia/reoxygenation (H/R).

**Results:**

*In vivo*, didymin reduced the pathological damage and fibrosis of myocardial tissues, decreased the levels of lactate dehydrogenase, creatine kinase, connective tissue growth factor, collagen I and collagen III. Moreover, didymin reduced myocardial apoptosis, inhibited NLRP3, ASC and caspase-1 expression, and alleviated the inflammatory response. *In vitro*, didymin reduced MI, apoptosis, inflammation and the levels of NLRP3, ASC and caspase-1 in H9C2.

**Discussion and conclusions:**

Didymin prevented the deterioration of MI by inhibiting NLRP3 inflammasome *in vivo* and *in vitro*, and may be a potential natural drug for the treatment of MI. Our study provides the scientific basis for further research of didymin.

## Introduction

Globally, the morbidity and mortality of cardiovascular diseases remain high (Meng et al. 2018). Most of the patients who die of cardiovascular diseases are due to myocardial infarction (MI), and the most fatal factor of MI is insufficient blood supply to the heart (Neri et al. 2017). Clinically, thrombolytic/fibrinolytic therapy and percutaneous coronary intervention can effectively restore the blood flow of ischaemic myocardium (Bagheri et al. 2016; Khan et al. 2018). However, reperfusion by the above methods may result in ischaemia–reperfusion (I/R) (Sun G et al. 2016). According to reports, various pathophysiological factors are involved in I/R injury, such as calcium overload, endothelial dysfunction, oxygen free radical production, immune response, mitochondrial dysfunction and myocardial apoptosis (Matsui et al. 2007; Mokhtari-Zaer et al. 2018). Currently, although major advances have been made in the mechanisms and resolution strategy of I/R injury, the protective function of clinical therapies appears to be limited. Therefore, understanding other molecular events related to I/R injury and finding new targets to reduce I/R injury are still urgently needed.

Due to numerous biological and pharmacological functions, flavonoids have aroused increasing interest in the discovery of drugs (Parhiz et al. 2015). Didymin is a naturally occurring flavonoid glycoside found in various citrus fruits, such as mandarins, oranges and lemons (Wei et al. 2017). Traditionally, didymin has long been used as a dietary antioxidant (Ali et al. 2019). In recent years, more and more evidence has shown that didymin exhibited a wide range of biological activities, including anticancer, anti-inflammatory, liver and vascular protection. For example, Shukla et al. (2018) proved that didymin effectively prevented hyperglycaemia-induced endothelial dysfunction and death by exerting antioxidant and anti-inflammatory effects.

Hung et al. (2010) described didymin by modulating Fas/Fas L apoptotic signal to inhibit the proliferative activity of non-small-cell lung cancer cells. In terms of liver protection, didymin ameliorated hepatic inflammation and hepatocyte apoptosis in mice non-alcoholic fatty liver by inhibiting the TLR4/NF-κB and PI3K/Akt pathways (Feng et al. 2020). Despite extensive data on didymin’s functional diversity, the potential use of didymin in the treatment of MI is not well known. In this research, we study the role and mechanism of didymin against MI through animal tissues and cell models. Our data will bring new prospect for the treatment of MI.

## Materials and methods

### Animals and groups

C57/BL6 mice (aged 6–8 weeks) were purchased from Vital River (Beijing, China). Didymin (purity >98%, #B21364) was supplied by Shyuanye (Shanghai, China). The mice (*n* = 40) were divided into five groups: sham group, I/R group, I/R + didymin (1 mg/kg) group, I/R + didymin (2 mg/kg) group and I/R + didymin (4 mg/kg) group. Didymin was administered intragastrically daily before I/R for five consecutive days. This study was conducted in accordance with the guidelines of Laboratory Animal Care and Use of Laboratory Animals and was approved by the Ethics Committee of First Affiliated Hospital, School of Medicine, Shihezi University (protocol number: SHZDW20210603).

### I/R model

After the last administration, mice were first anaesthetized with sodium pentobarbital (50 mg/kg, ip). Then, the thoracotomy was performed to expose the heart. The left coronary artery (LCA) was ligated and bound with 7-0 nylon monofilament for 30 min to induce ischaemia. After occlusion for 30 min, the nylon monofilament was released and reperfused was performed for 2 h to obtain I/R injury model.

### Cell culture and treatment

Rat cardiomyocytes (H9C2 cells) were obtained from Nanjing Keygen Biotech (Nanjing, China). After all the cells were resuscitated, they were cultured in DEME medium. The cells were divided into five groups: control group, H/R group, H/R + didymin (3 μM) group, H/R + didymin (10 μM) group and H/R + didymin (30 μM) group. Didymin was administered 24 h before hypoxia/reoxygenation (H/R).

### Hypoxia/reoxygenation model

Briefly, H9C2 cells with a confluence rate of 80% were first cultured in the glucose-free DEME medium with 95% air and 5% CO_2_. After 48 h, the cells were then incubated for 3 h at 37 °C in an incubator containing 94% N_2_, 5% CO_2_ and 1% O_2_. Finally, the cells were oxygenated for 6 h at 37 °C in 95% air and 5% CO_2_.

### Echocardiography and haemodynamics indexes

First, the mice were anaesthetized with sodium pentobarbital (50 mg/kg, ip) and placed on the plate supine. Then, the acupuncture needles were inserted in the limbs and connected to the leads. Finally, a Vevo2100 high-frequency ultrasound system (Visual Sonics Inc., Toronto, Canada) was used to collect the ultrasound images. For the ultrasonic index detection, the left ventricular ejection fractions (LVEFs) and left ventricular fractional shortening (LVFS) were calculated. For hemodynamic detection, the maximum rate of rise of left ventricular pressure increase (+d*p*/d*t*_max_) and the maximum rate of rise of left ventricular pressure decrease (–d*p*/d*t*_max_) were recorded.

### Measurement of LDH, CK, IL-18, IL-1β and TNF-α

The LDH and CK level in the supernatant of tissues homogenate and cells culture was detected using the LDH assay kit (#A020-2-1, Jiangcheng, Nanjing, China) and the CK assay kit (#A032-1-1, Jiangcheng, Nanjing, China). Moreover, the IL-18, IL-1β and TNF-α level in the supernatant of tissues homogenate was determined by using the commercial kits (IL-18, #ml002294, Mlbio, Shanghai, China; IL-1β, #PI301, Beyotime Biotechnology, Beijing, China; TNF-α, #ml002095, Mlbio, Shanghai, China).

### H&E staining

After fixed with formaldehyde (10%), the tissues samples were cultured in a 5% nitric acid decalcification solution for 3–4 d. Then, the tissue sections were subjected to routine dehydration, transparency, paraffin immersion, embedding and sectioning. Finally, the samples were stained with H&E, and histopathological changes were observed under a microscope (DP73; Olympus, Tokyo, Japan).

### Masson staining

First, slices of myocardial tissue were fixed in Bouin’s solution. After washing, the slices were stained in Mayer’s haematoxylin, acidic ponceau and aniline blue for 20 min, respectively. Finally, the samples were rapidly dehydrated in 95% ethanol and then treated with hyaluronic acidification with dimethylbenzene.

### TUNEL staining

First, the myocardial tissues were conventionally fixed, dewaxed, embedded and sectioned. After washing by PBS, the slices were added into TUNEL mixture (Beyotime Biotechnology, Beijing, China) for incubation. Subsequently, the sections were further stained with DAPI (Beyotime Biotechnology, Beijing, China) and finally observed under a microscope.

### qRT-PCR

After the RNA was isolated and prepared, the expression level was detected with FAST SYBRTM Green Master Mix. β-actin was set as internal parameter. The primer sequences for this experiment were as follows: IL-18 sense 5′-TCAGACAACTTTGGCCGACT-3′ and antisense 5′-CAGGTGGATCCATTTCCACTTTG-3′; IL-1β sense 5′-AAATGCCACCTTTTGACAGTGATG-3′ and antisense 5′-GCAGCCCTTCATCTTTTGGG-3′; TNF-α sense 5′-GTCCCCAAAGGGATGAGAAGT-3′ and antisense 5′-TTTGCTACGACGTGGGCTAC-3′; β-actin sense 5′-ATATCGCTGCGCTGGTCG-3′ and antisense 5′-TTCCCACCATCACACCCTGG-3′.

### Western blot

The target cell protein was obtained by RIPA method, and the protein concentration was determined by BCA kit (Solarbio, Beijing, China). Then, prepared protein was taken for gel electrophoresis separation, and the isolated protein was electrically transferred to PVDF membrane. After blocked with 5% skim milk, the PVDF membrane was subjected to primary antibodies: connective tissue growth factor (CTGF, #ab6992, 1:1000, Abcam, Cambridge, UK), collagen I (#ab270993, 1:1000, Abcam, Cambridge, UK), collagen III (#ab7778, 1:1000, Abcam, Cambridge, UK), Bcl-2 (#ab196495, 1:1000, Abcam, Cambridge, UK), Bax (#ab32503, 1:1000, Abcam, Cambridge, UK), NOD-like receptor protein 3 (NLRP3, #ab263899, 1:1000, Abcam, Cambridge, UK), ASC (rat: #ab180799, 1:1000, Abcam, Cambridge, UK; mouse: #67824S, Cell Signaling, Danvers, MA), caspase-1 (#83383, 1:1000, Cell Signaling, Danvers, MA), IL-β (#ab254360, 1:1000, Abcam, Cambridge, UK), IL-18 (#ab191860, 1:1000, Abcam, Cambridge, UK) and GAPDH (#ab9485, 1:1000, Abcam, Cambridge, UK). The next day, the PVDF membrane was incubated with secondary antibody (#ab288151, 1:5000, Abcam, Cambridge, UK) at room temperature. An enhanced chemiluminescence kit (ECL, #P0018S, Beyotime Biotechnology, Beijing, China) was added and exposed in the gel imaging system. The protein content was analysed using Quantity-One software (Bio-Rad, Hercules, CA).

### MTT assay

In brief, H9C2 cells were cultivated in 96-well plates. Subsequently, 20 μL of MTT solution (#ST316, Beyotime, Shanghai, China) was complemented to per well. After hatched for 4 h, 100 μL DMSO was added. Ultimately, the absorbance was monitored at 570 nm using a microplate reader (Thermo Scientific, Waltham, MA).

### Flow cytometry

In short, collected H9C2 cells were re-suspended with binding buffer. Then, the samples were stained with Annexin V and PI (Beyotime Biotechnology, Beijing, China) for 30 min avoid of light. After washing, cell apoptosis of treated cells was measured by a flow cytometer (BD Biosciences, Franklin Lakes, NJ).

### Statistical analysis

Data from this research were analysed by SPSS 20.0 (SPSS Inc., Chicago, IL). The differences between groups were calculated via one-way ANOVA followed by Dunnett’s multiple comparison. *p*< 0.05 means there is a statistical difference.

## Results

### Didymin relieved myocardial infarction injury after I/R

**Figure 1.**
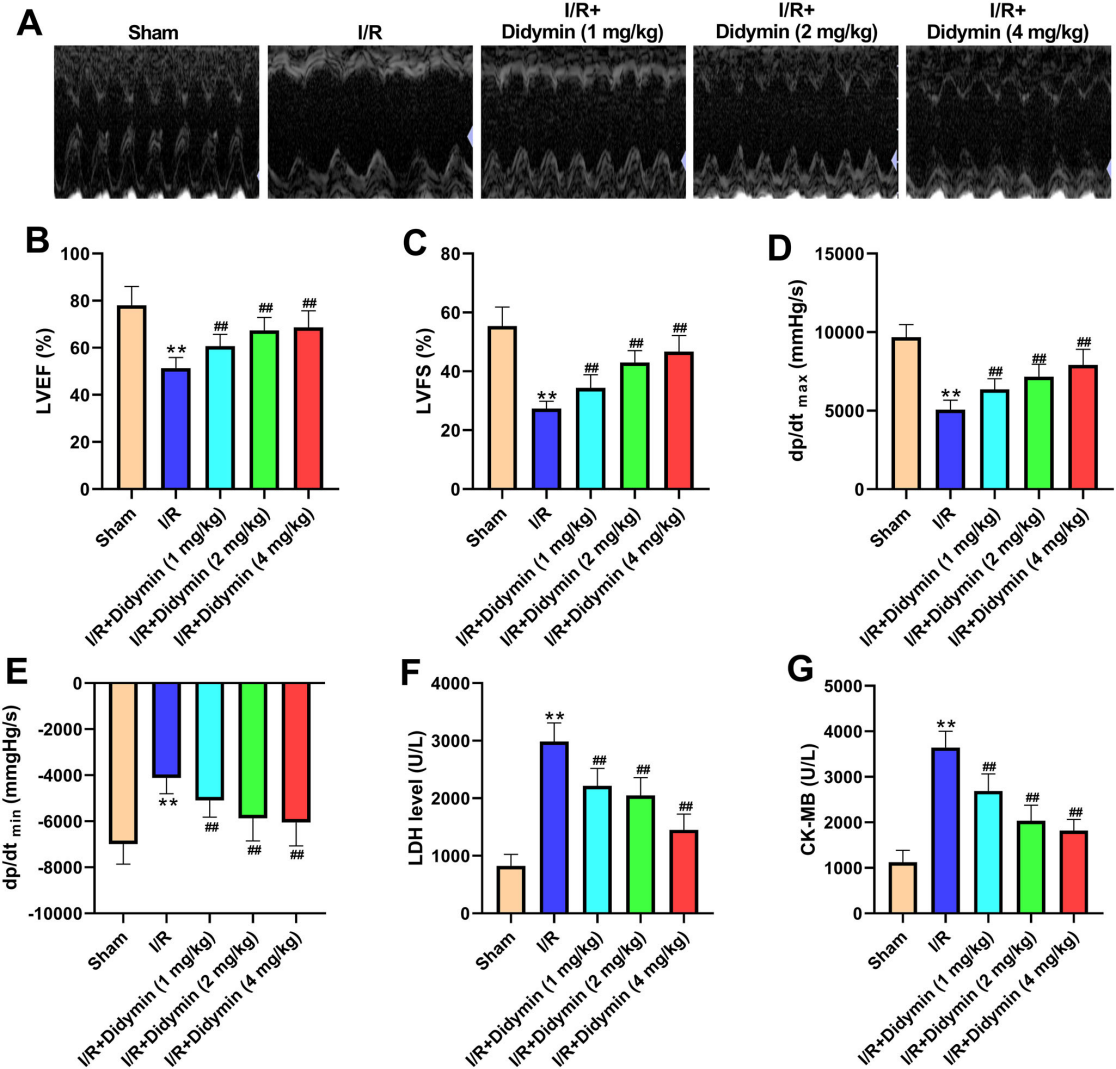
Didymin relieved myocardial infarction injury after I/R. (A) The echocardiography of mice in indicated groups; (B, C) the change of ultrasonic indexes; (D, E) the change of haemodynamics indexes; (F, G) the levels of LDH and CK in myocardial tissue. **p < 0.01 vs. sham group; ^##^*p* < 0.01 vs. I/R group.

Echocardiography data showed that compared with the I/R group, the myocardial state of mice in the I/R + didymin group was sharply increased (Figure 1(A)). Further, ultrasonic indexes and haemodynamics indexes were monitored and there were differences between groups. As depicted in Figure 1(B–E), LVEF, LVFS and d*p*/d*t*_max_ were significantly reduced and d*p*/d*t*_min_ increased compared with sham group, while reversed after didymin treatment compared with I/R group. Meanwhile, LDH and CK in serum, which are typical markers of myocardial injury, also decreased significantly after different concentrations of didymin intervention (Figure 1(F,G)).

### Didymin relieved cardiac fibrosis caused by myocardial infarction

**Figure 2.**
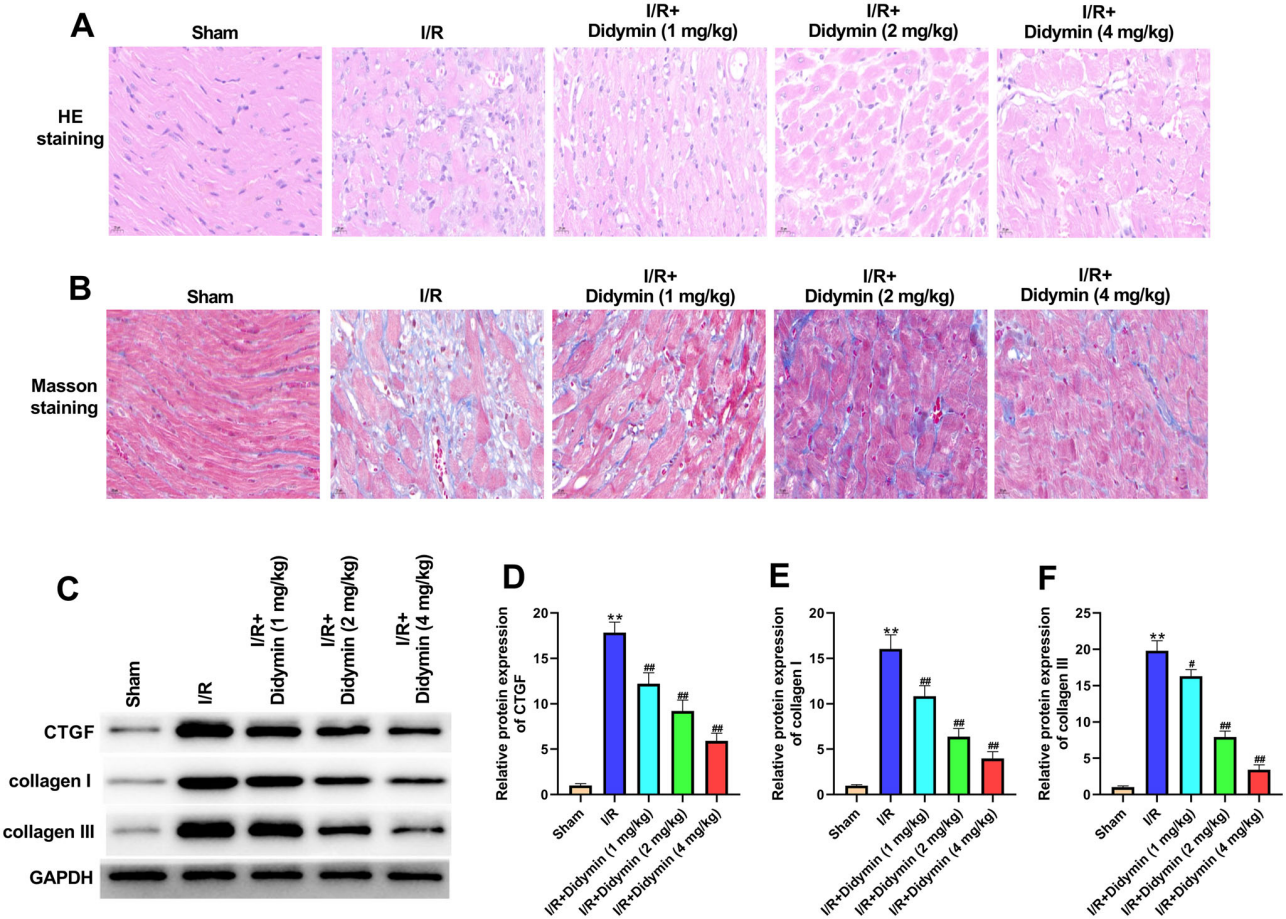
Didymin relieved cardiac fibrosis caused by myocardial infarction. (A) H&E staining of myocardial tissues; (B) Masson staining of myocardial tissues; (C–F) Western blot was used to detect fibrotic factors in myocardial tissue. **p < 0.01 vs. sham group; ^#^*p* < 0.05, ^##^*p* < 0.01 vs. I/R group.

To further examine the protective effect of didymin on myocardium, we observed the morphological changes of myocardial tissues. H&E staining data revealed that the myocardial tissues of sham group had clear structure and normal arrangement without pathological changes. In the I/R group, myocardial tissues were disordered, myocardial cells were enlarged and myocardial fibres were damaged. In the didymin group, the myocardial tissues were relatively clear, and myocardial damage was reduced (Figure 2(A)). For Masson staining, the myocardial fibre arrangement was disordered and collagen deposition was abundant in the I/R group. Interestingly, however, didymin evidently reduced the accumulation of collagen fibres between cardiac muscle cells as comparison to I/R group (Figure 2(B)). In addition, we further examined the expression of markers that reflect the degree of myocardial fibrosis. Western blot evidence showed that CTGF, collagen I and collagen III protein levels were evidently increased in the I/R group. Instead, didymin reduced the expression of above-mentioned proteins as compared to I/R group (Figure 2(C–F)).

### Didymin reduced apoptosis induced by myocardial infarction

**Figure 3.**
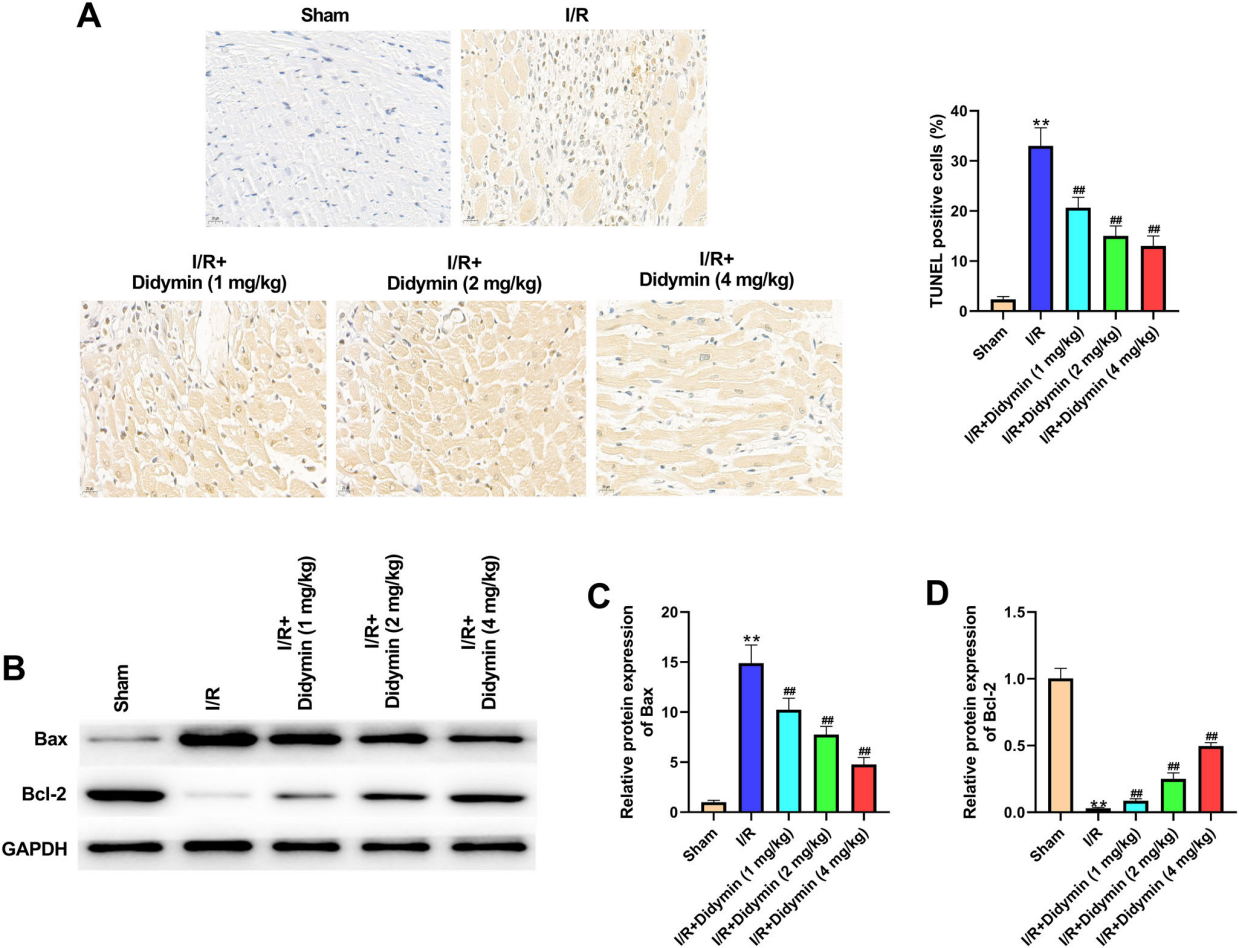
Didymin reduced apoptosis induced by myocardial infarction. (A) TUNEL staining of myocardial tissues; (B–D) Western blot was used to measure the levels of apoptosis factors in myocardial tissue; **p < 0.01 vs. sham group; ^##^*p* < 0.01 vs. I/R group.

Compared with sham group, TUNEL positive cells were remarkably increased in I/R group. However, after didymin administration, TUNEL positive cells were significantly reduced (Figure 3(A)). Next, we analysed the expression of apoptosis-related proteins in each group. As expected, I/R injury significantly induced Bax expression and reduced Bcl-2 expression. Meanwhile, we observed that didymin reversed the above-mentioned protein variation, manifested by a decrease in Bax protein and an increase in Bcl-2 protein (Figure 3(B–D)).

### Didymin relieved inflammation caused by myocardial infarction

**Figure 4.**
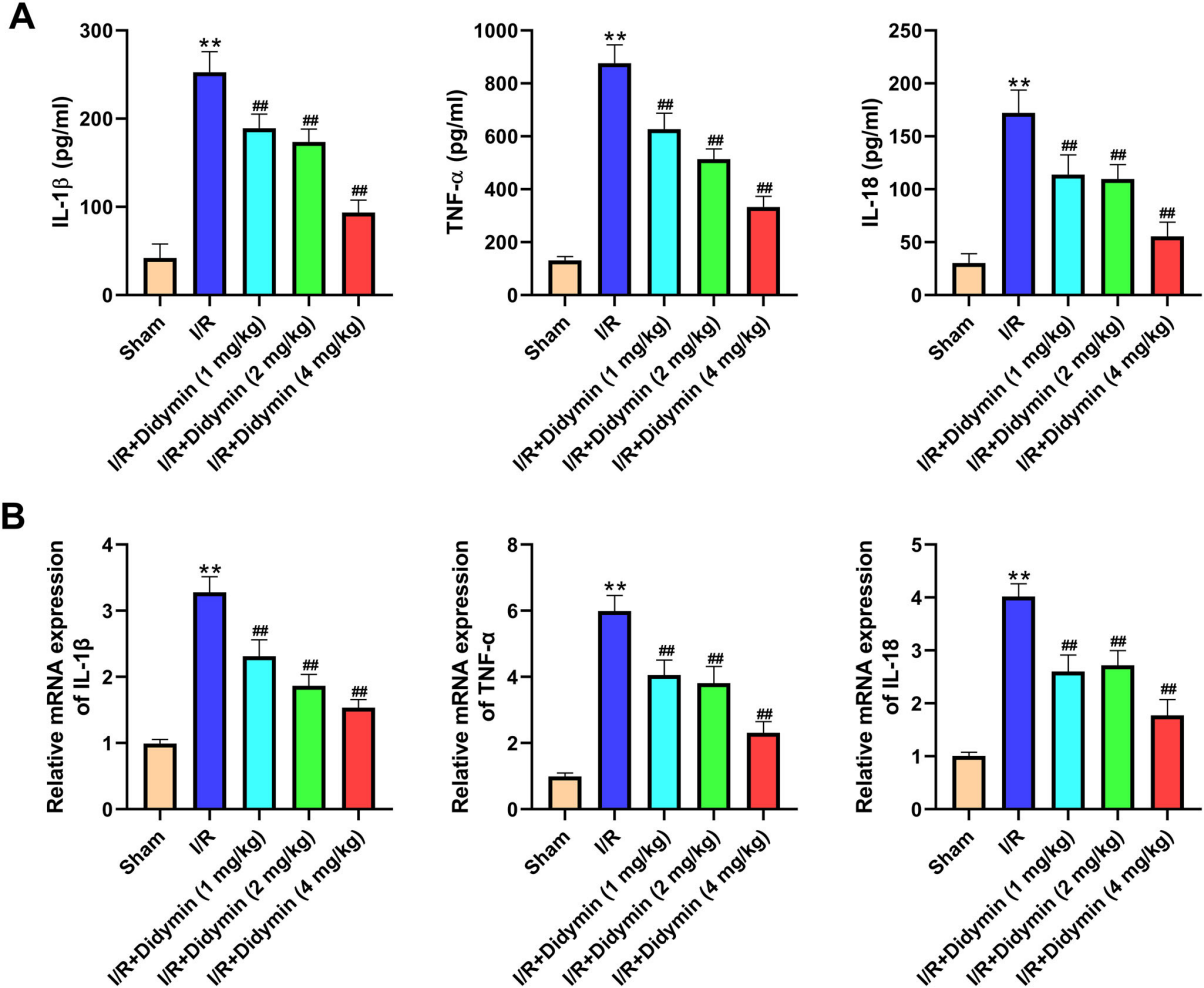
Didymin relieved inflammation caused by myocardial infarction. (A) The levels of inflammatory factors in myocardial tissue were measured by ELISA; (B) PCR was used to measure inflammatory factors in myocardial tissue. **p < 0.01 vs. sham group; ^##^*p* < 0.01 vs. I/R group.

Next, we assessed the influence of didymin on the inflammatory response. As shown in Figure 4, the contents of IL-1β, IL-18 and TNF-α in serum were significantly increased after I/R in mice compared with sham group. After the intervention of didymin, the levels of IL-1β, IL-18 and TNF-α in the tissue homogenate were remarkably reduced. In addition, we also detected mRNA changes of the three inflammatory factors mentioned above. Consistent with the above results, we observed that I/R evidently induced high expression of IL-1β, IL-18 and TNF-α. Conversely, didymin reduced the high levels of IL-1β, IL-18 and TNF-α as comparison to I/R group (Figure 4(B)).

### Didymin relieves myocardial infarction by inhibiting NLRP3 inflammasome

**Figure 5.**
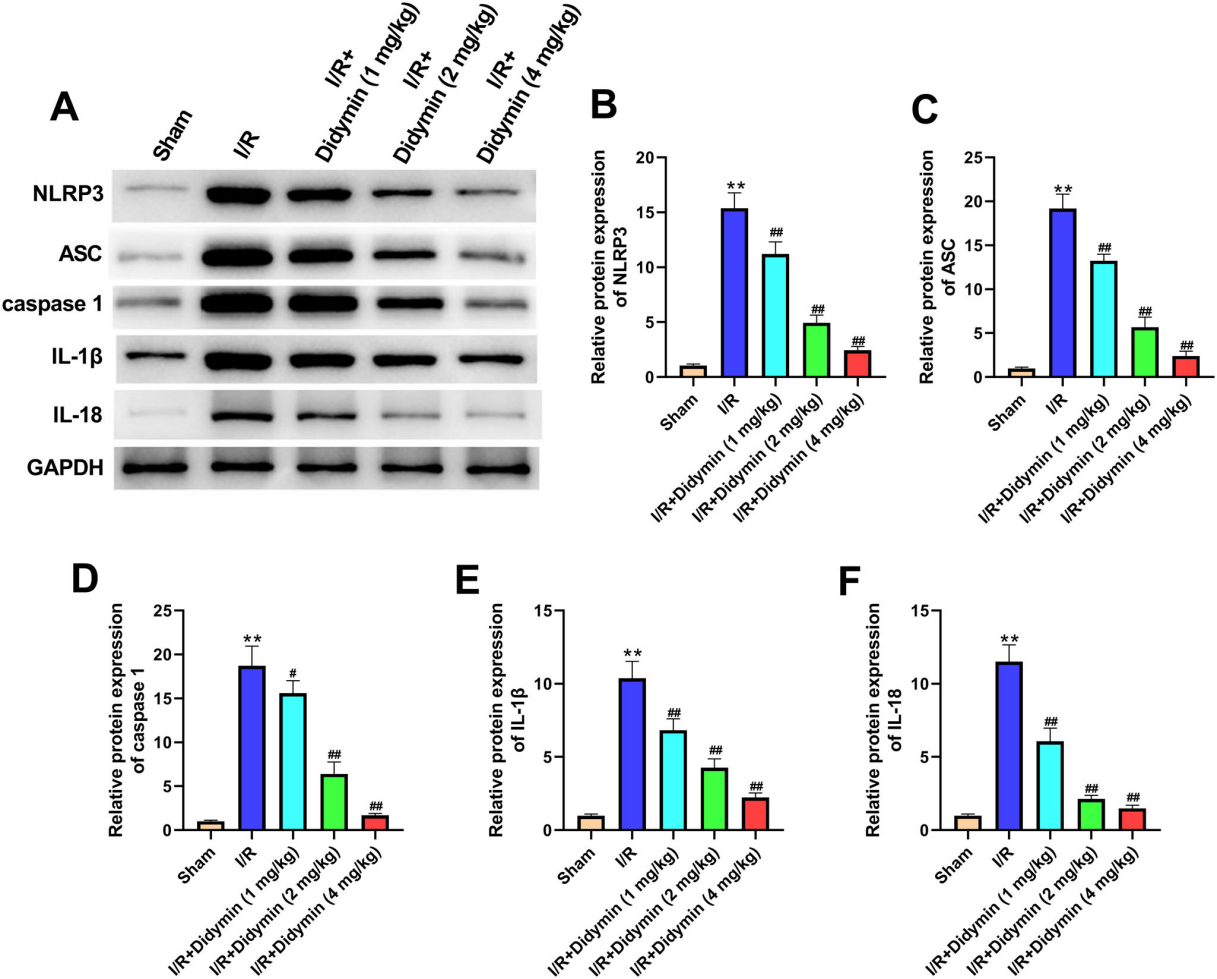
Didymin relieves myocardial infarction by inhibiting NLRP3 inflammasome. (A–F) The expression of NLRP3, ASC, caspase-1, IL-1β and IL-18 in myocardial tissue was determined by western blot. **p < 0.01 vs. sham group; ^#^*p* < 0.05, ^##^*p* < 0.01 vs. I/R group.

Western blot finding showed that I/R significantly increased the expression of proteins NLRP3, ASC, caspase-1, IL-1β and IL-18 (Figure 5(A–F)). Meanwhile, the elevated expressions of NLRP3, ASC, caspase-1, IL-1β and IL-18 were remarkably reversed by the treatment of didymin (Figure 5(A–F)). All these results suggested that didymin could relieve MI by inhibiting NLRP3 inflammasome *in vivo*.

### Didymin alleviates H/R induced cardiomyocyte injury by inhibiting NLRP3 inflammasome

**Figure 6.**
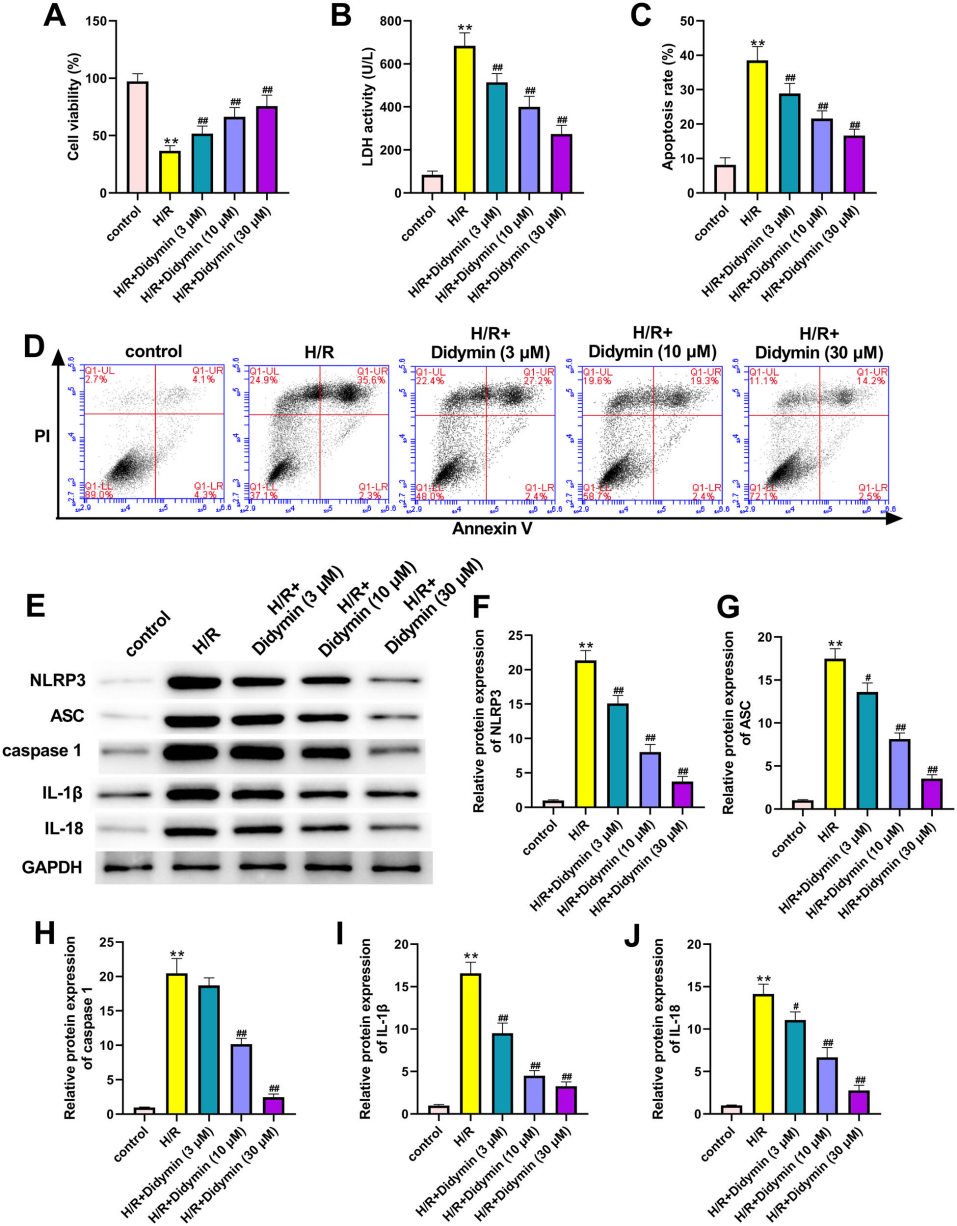
Didymin alleviates H/R induced cardiomyocyte injury by inhibiting NLRP3 inflammasome. (A) Cell viability was measured by MTT; (B) the LDH level in H9C2 was detected via commercial kits; (C, D) cell apoptosis was detected by flow cytometry; (E–J) the expression of related proteins was detected by western blot. **p < 0.01 vs. control group; ^#^*p* < 0.05, ^##^*p* < 0.01 vs. H/R group.

To further explore the effect of didymin on NLRP3-dependent H/R injury *in vitro*, H9C2 cells were treated with different concentrations of didymin and found that didymin significantly reversed the injury effect of H/R on cells. Specifically, we found that didymin significantly increased cell activity, decreased LDH activity and reduced myocardial cell apoptosis (Figure 6(A–D)). Moreover, compared with the H/R group, didymin significantly inhibited NLRP3 activation and downstream expression of related proteins, including ASC, caspase-1, IL-1β and IL-18 (Figure 6(E–J)).

## Discussion

Clinically, secondary injury such as I/R injury will further lead to arrhythmia, myocardial tissue necrosis, myocardial dysfunction and other abnormal symptoms (Liang et al. 2021). After I/R, the myocardial function of patients was significantly reduced, manifested by decreased LVEF and LVFS, increased diastolic and systolic inner diameters, increased ventricular volume and decreased cardiac output (Bajpai et al. 2019; Yu et al. 2021). Here, we found that didymin significantly increased LVEF, LVFS and d*p*/d*t*_max_, while significantly decreased d*p*/d*t*_min_, which was consistent with previous reports. LDH and CK are special enzymes present in myocardial tissues, and their levels are positively correlated with the degree of myocardial injury (Xu et al. 2019; Yao et al. 2019). Also in our study, we observed that the didymin evidently reduced the expression of LDH and CK induced by I/R. In summary, our data reveal that didymin significantly improved myocardial function, thereby effectively reducing I/R.

Myocardial fibrosis is a common pathological manifestation of myocardial injury to a certain extent and is the most characteristic structural change (Rathinavel et al. 2018). Therefore, fibrotic repair after MI is of irreplaceable significance in maintaining normal myocardial function. CTGF is an important inflammatory factor promoting myocardial fibrosis, which can promote collagen synthesis and external matrix precipitation (Sun et al. 2017). Type I and type III collagen are the main proteins involved in fibrosis, and their excessive deposition will affect the generation and transmission of force between myocardial cells, and in severe cases can lead to heart failure (Talman and Ruskoaho 2016). In our study, we found that I/R significantly induced the expression of these proteins. In the process of seeking treatment for fibrosis, Huang et al. (2018) proved that liraglutide can improve myocardial fibrosis after MI by inhibiting the expression of CTGF. Yu et al. (2020) showed that Elabela alleviated I/R induced fibrosis by inhibiting collagen precipitation. Excitingly, in this study, didymin was confirmed to reduce I/R-induced myocardial fibrosis, accompanied by the decrease of CTGF, collagen I and collagen III, indicating that didymin was also a potential drug for the treatment of myocardial fibrosis. As we all know, apoptosis is an important sign of I/R injury, and cell apoptosis increased during I/R (Aghaei et al. 2019). Consistently, our *in vitro* and *in vivo* data both suggested that I/R increased myocardial apoptosis. Meanwhile, we also confirmed that the addition of didymin affected apoptosis-related expression, accompanied by a decrease in Bax and an increase in Bcl-2, which was consistent with the Zhou et al. (2020) results that salvianolic acid A inhibited I/R-induced apoptosis by down-regulating Bax/Bcl-2. Collectively, the above data suggested didymin significantly alleviated I/R induced myocardial fibrosis and apoptosis.

A large amount of data showed that inflammation can aggravate myocardial I/R injury, so many pharmacological studies are focussed on inhibiting the inflammatory response in exploring the reduction of I/R injury (Rameshrad et al. 2016; Kosuru et al. 2018). For example, Bai et al. (2019) demonstrated that biochanin A alleviated I/R by reducing the expression of inflammatory cytokines IL-1β and IL-18. Consistently, we also found that didymin reduced the levels of the above-mentioned inflammatory factors in myocardial tissue and cells. NLRP3 inflammasome is a participant of inflammatory immune response, consisting of NLRP3, ASC and pro-caspase-1 precursor, which has been confirmed to be involved in the occurrence and development of myocardial I/R injury (Luo et al. 2017; Yue et al. 2019). After NLRP3 is activated, it binds to ASC and further activates caspase-1. Then, the activated caspase-1 continues to cleave the precursors of IL-1β and IL-18 as active cytokines, which are involved in the development of inflammation and myocardial I/R injury. Kawaguchi et al. (2011) demonstrated that NLRP3 was activated and promoted inflammatory cytokine release during I/R. In addition, Wang et al. (2020) reported that artemisinin alleviated myocardial injury by inhibiting NLRP3 inflammasome activation. As expected, our data suggested that NLRP3, ASC, IL-1β and IL-18 expression increase after I/R injury, while didymin treatment inhibited NLRP3 inflammasome activation and inflammatory response.

## Conclusions

In this study, we examined the effect of didymin on MI *in vitro* and *in vivo*. The results showed that didymin can improve myocardial dysfunction after I/R, inhibit myocardial fibrosis, apoptosis, inflammation and NLRP3 activation. Therefore, didymin can be developed as a potential therapeutic drug for MI.

## Data Availability

The datasets used and analysed during the current study are available from the corresponding author.
